# Perceptions and opinions of Nigerians to the management and response to COVID-19 in Nigeria

**DOI:** 10.11604/pamj.2021.40.185.31824

**Published:** 2021-11-26

**Authors:** Obinna Ositadimma Oleribe, Ifeoma Eugenia Idigbe, Princess Osita-Oleribe, Olatayo Olawepo, Zaidat Adesola Musa, Samuel Aikhuomogbe, Oliver Chukwujekwu Ezechi, Michael Fertleman, Babatunde Salako, Simon David Taylor-Robinson

**Affiliations:** 1 Family Services Department, Klamath Tribal Health and Family Services, Klamath Falls, Oregon, United States of America,; 2 Department of Public Health, Nigeria Institute for Medical Research, PMB 2013, Yaba, Lagos, Nigeria,; 3 Office of the Director, Center for Family Health Initiative, Kubwa, Abuja, Nigeria,; 4 Office of the Central Secretariat, Georgetown Global Health, Abuja, Nigeria,; 5 Department of Public Health, Nigeria Institute for Medical Research, PMB 2013, Yaba, Lagos, Nigeria,; 6 Baobab Group, 360, Murtala Muhammed Way, Yaba, Lagos 2013, Nigeria,; 7 Office of the Central Secretariat, Nigeria Institute for Medical Research, Yaba, Lagos, Nigeria,; 8 Cutrale Perioperative and Ageing Group, Department of Bioengineering, Imperial College London, White City Campus, 86 Wood Lane, London W120BZ, United Kingdom,; 9 Office of the Director General, Nigeria Institute for Medical Research, PMB 2013, Yaba, Lagos, Nigeria,; 10 Department of Surgery and Cancer, Imperial College London, London W21NY, United Kingdom

**Keywords:** COVID-19, Nigeria, public perceptions

## Abstract

**Introduction:**

we present a qualitative analysis of opinions of the Nigerian general public as to how successful healthcare strategies have been in containing the COVID-19 outbreak.

**Methods:**

an online qualitative survey was conducted, consisting of 30 semi-structured questions.

**Results:**

four hundred and ninety-five (495) respondents participated, ranging in age from 18 to 59 years. Over 40% of all respondents were critical of public health information. Participants saw provision of social support measures (n = 83), lack of economic, financial and social support (n = 65), enforcement of restrictions on movement outside the home, availability of face-masks and social distancing (n = 53) and provision of COVID-19 testing (n = 48) as the major things that were handled poorly by the government and health authorities.

**Conclusion:**

we advocate coordinated forward planning for public safety until vaccines are widely available; while social distancing should continue. Policymakers need to be adaptable to changing conditions, given fluctuating case numbers and fatality rates.

## Introduction

COVID-19 is still a new global phenomenon and like every new challenge, it is fraught with management and containment challenges. As of October 15^th^ 2021, according to the World Health Organization (WHO), there were 239,437,517 confirmed cases and 4,879,235 COVID-19-related deaths around the world with a case fatality rate (CFR) of 2.0% [1]. Of these, Africa has had 6,102,089 confirmed cases with 148,789 deaths and a slightly higher CFR of 2.4% [1].

As a new disease, new practices, policies and procedures were developed to manage the pandemic around the world [2]. Under WHO aegis, most nations developed or adopted management protocols quickly and put these into use without prior consideration of their effectiveness in handling the disease outbreak in different communities. Some policies, such as social distancing, hand washing, “stay at home” orders and self-isolation/quarantine measures were adopted almost uniformly without thought on their applicability in different cultures, where education on modern disease concepts were rudimentary or absent [3]. Such measures were implemented rapidly without thought as to their economic consequences or on societal mental health and well-being in areas of the world where there was little concept as to what a “virus” may be [3].

Since February 27^th^, 2020 when the first case was identified in Nigeria, the Federal Ministry of Health (FMOH), and its key agencies, such as the Nigerian Center for Disease Control (NCDC) have implemented WHO recommendations towards the control of COVID-19 in their entirety. NCDC has also adopted and adapted various policies, treatment guidelines and standards of practice from previous local epidemics, such as Ebola, while opening isolation and treatment centres, assessing and approving several laboratories for COVID-19 diagnosis, in addition to training/retraining healthcare workers to manage the COVID-19 outbreak [3]. Despite these major mile-stones, the outbreak has expanded from one case in February 27^th^, 2020 to 164,233 confirmed and documented cases as of April 19^th^, 2021 with 2,061 deaths and a CFR of 1.3% [4].

Nigeria is a country used to controlling outbreaks of highly infectious viral infections successfully, including Lassa fever, Ebola and Marburg virus diseases, and given the low death rates, it would appear that there has been some success with COVID-19 transmission [4]. However, there is a need to assess the applicability of COVID-19 policies in Nigeria, Africa´s most populous country and a potential hotbed of disease transmission. Previous studies have focused on how consumer attitude impacts consumer preventive equipment purchase intention, and the effect of boredom from limited daily activities due to long-term quarantine [5, 6]. In this context, our qualitative study was aimed at assessing the applicability of COVID-19 preventive measures taken by an online survey of a sample of the Nigerian population.

We originally performed a quantitative survey on public opinions in 2020 [7], but in order to further assess the applicability of the COVID-19 containment measures taken since that time, we conducted a follow-up online qualitative survey of this representative sample of the Nigerian population to identify what was perceived to have gone well with COVID-19 public health measures, what was perceived not to have gone well, and what in the opinion of those surveyed could be changed to improve the outbreak control process and outcomes, in addition to what could be gleaned in preparation for future outbreaks.

## Methods

**Timeline:** this qualitative study was conducted in March 2021, through an anonymous online cross-sectional survey. The original quantitative study was performed in 2020 [7]. The same participants took part in both studies, but on this occasion, they were invited to respond in open prose, rather than to rate responses in a quantitative manner.

**Participants:** participants were originally recruited by online advertisement using a self-selecting sampling technique. The participants received a “one time only” link to the online questionnaire. They were informed of the anonymity of their participation for publication purposes and that the information collected would be kept confidential. Only participants 18 years and above were invited to participate in the survey, but apart from that there were no other selection criteria or stipulations on recruitment. Demographic and contact data were kept behind a computer firewall to comply with international General Data Protection Regulations (GDPR) regulations. As a follow-up to the original quantitative study [7], all the original subjects who participated were contacted by email and/or by WhatsApp one year after the original study with additional questions to assess whether new information could be gleaned through open prose, rather than quantitative rating of closed questions.

**Sample size:** we calculated an approximate minimum sample size of 452 at 5% precision, 95% confidence interval, and 50% response distribution (online survey).

**Study design:** this was a qualitative study in its design. Consolidated criteria for reporting qualitative research (COREQ) guidelines were followed. The qualitative collected was fully transcribed into a Microsoft Word™ (Microsoft, Redmond, Washington, USA) text document and the context organised into themes for thematic analysis by each of the two authors who acted independently. Interrater reliability was greater than 70%, using Cronbach´s alpha test. A STROBE checklist was also completed.

**Data collection procedure:** Google Form was used to develop and distribute the questionnaire. The questionnaire was in English and was pretested for accuracy, cultural sensitivity, comprehensibility and analysability before the study was commenced. Initial invitations to participate in the original quantitative study were through private messages, social groups, and several social networking sites and platforms [7]. Personal and group emails, WhatsApp and Facebook messages were subsequently used to contact participants after the initial expression of interest. A “one time only” link to the online questionnaire prevented multiple survey completion from the same participant.

Invitations to take part in this follow-up survey were by direct email or WhatsApp reminder to the original participants and like the original quantitative study, the links were of “one time only” format. By clicking on this “one time only” link to the questionnaire, the participant was directed to the survey's entry page, which contained information on the objectives of the survey, terms of participation, and data privacy. Participants were informed about the possible risks and benefits of the survey. They were then asked to complete the survey in one session, which took between 10 to 15 minutes. Failure to do so in one sitting resulted in the loss of the “one time only” link to the questionnaire. For ease of access, participants were able to access the survey and complete it either on a computer or a mobile device.

The current survey consisted of 30 semi-structured questions. The questionnaire collected information on sociodemographic characteristics, knowledge of COVID-19 and individual perspective on management and response to the pandemic. The questions were semi structured, with some questions allowing participants to provide detailed written answers to obtain a more profound sense of their perspective. The data collected for the survey and utilised in this study were stored electronically and were password protected.

### Measures

**Response to COVID-19:** participant perception of the measures introduced by the Federal Government and Governmental institutions to the COVID-19 pandemic were assessed in the survey.

**Data analysis:** data collected on Google Analytic Tool were exported and analysed with MS Excel sheets (Microsoft Inc., Redmond, Washington, USA). Questions were analysed using thematic analyses by a team of the first three authors acting together. The responses were categorised in themes, entered into a code book and harmonized by two independent researchers. Using the themes developed, salient responses were highlighted, explored and discussed to depict the responses. Results are provided in tabular format for information on response characterisation and in narrative fashion for the various comments made.

**Ethics:** prior ethics committee approval for this anonymised survey was obtained from the local research ethics committee of the Nigerian Institute for Medical Research (NIMR) in April 2020 (reference NIMR EC 2020-62G). The study was conducted in line with the precepts set out in the 1975 Declaration of Helsinki. All participants consented to their anonymous responses being collated and used for research purposes. Demographic data were anonymised and stored in anonymised form behind a computer security firewall to comply with GDPR regulations.

## Results

The exact same 495 respondents who participated in this study, took part in the original quantitative study [5], of which 482 (97.4%) respondents were resident in Nigeria. The respondents ranged from 18 to 59 years, with a mean age of 42.1 ± 9.7 years. Most of the respondents were married (76.6%), were males (61.8%), had tertiary level education (91.0%), were public servants (36.8%), Christians (82.6%), and resident either in the Federal Capital Territory (49.1%) or the South-East region of the country (36.6%). 98.8% of the respondents had heard of COVID-19 (n = 489), and knew it that it was a viral disease (n = 472; 95.4%). The demographic data are tabulated in Table 1.

**Table 1:** participant responses on what was not conducted satisfactorily in the management and control of the COVID-19 pandemic in Nigeria from February 2020

Characteristics	Respondents, n, (%)
**Country of residence**	
Nigeria	482 (97.4)
Others	13 (2.6)
**Geopolitical zone of residence**	
North-Central	243 (49.1)
North-East	29 (5.9)
North-West	30 (6.1)
South-East	68 (13.7)
South-South	39 (7.9)
South-West	86 (17.4)
**Marital status**	
Single	100 (20.2)
Married	379 (76.6)
Divorced/separated	9 (1.8)
Widowed	7 (1.4)
**Age (years)**	
Less than 20	1 (0.2)
20-29	61 (12.3)
30-39	142 (28.7)
40-49	159 (32.1)
50-59	93 (18.8)
60 years and above	39 (7.9)
**Sex**	
Female	189 (38.2)
Male	306 (61.8)
**Religion**	
African traditional religion	2 (0.4)
Christianity	409 (82.6)
Islam	79 (16.0)
Others	3 (0.6)
**Education qualification**	
Non formal	18 (3.6)
Primary	2 (0.4)
Secondary	20 (4.0)
Tertiary	455 (91.9)
Work status	
Working	338 (87.5)
Not working	38 (7.7)
Student	24 (4.8)

In analysing the open-ended question, four themes emerged. The themes were: communication and creating awareness; enforcing safety precaution measures; government support in controlling COVID-19 and engaging major health stakeholders, while in the minority of respondents, according to the participants, the four top areas that were perceived by the respondents to have been conducted well in Nigeria included Governmental public health communication, awareness and publicity (respondent n = 114/495); enforcement of “stay at home” measures and the use of face masks (n = 42/495); Governmental support and control measures for the epidemic (n = 38/495) and NCDC COVID-19 management (n = 30/495). However, 92 respondents did not perceive anything that was conducted satisfactorily.

In expressing themselves on what went well, the respondents commented that:

“*Efforts continually made by the NCDC workers in managing the epidemic by testing individuals, spreading information about the epidemic via text messages*”;

“*The enforcement of compulsory face-masks*”;

“*The early awareness, using different mediums of communication and involving traditional leaders*”;

“*Closure of international borders and restriction of international flights and the setting up of isolation centers*”;

“`*Communication and information about the epidemic, prevention messages and support received by religious bodies, families and friends*”;

“*Improvements in personal hygiene*”;

“*Implementing social distancing and enforcement of stay at home measures, use of face-masks and hand-sanitizer and hand-washing policies*”;

“*Excellent publicity on COVID-19 and prevention of transmission*”;

“*Private sector contributions to the management of the COVID-19*.”

**Conversely:** the participants saw the provision of social support measures (n = 83/495); the availability of economic, financial and social support (n = 65/495); the enforcement of restrictions on movement outside the home, the availability of face-masks and social distancing (n = 53/495) and the provision of COVID-19 testing (n = 48/495) as the major things that were perceived not to have gone well (Table 1).

The respondents suggested that the performance of the Office of the Federal President (n = 106/495); outbreak update information given to the public (n = 30/495); management of dedicated healthcare resources and provision of testing equipment (n = 30/495) and the effectiveness of the Presidential Task Force on COVID-19 (n = 28/495) were the four aspects that were perceived in need of improvement by the respondents. Thirty-two respondents wanted everyone/everything involved in outbreak control changed.

Most participants said that the NCDC national COVID-19 strategy should be up-scaled and improved upon, the attitude and approach of the Federal Government towards governance should be made transparent, and communication methods should be clarified. Respondents also felt that enforcement messages should be made clearer totally public anxiety. Some other respondents believed that public opinion should be sought with respect to policy implementation. Others believed that lockdown procedures and their implementation should be improved and molecular testing should be diversified with all Federal Medical Centres (FMCs) having a laboratory dedicated to COVID-19. Also, respondents believed that there should be more community engagement, better provision of personal protective equipment (PPE) to front-line health workers, better distribution of social support, closer monitoring of border closures, and a halt to internal interstate movements within Nigeria during periods of high viral transmission.

Respondents suggested that in line with measures taken by other countries, the Federal Nigerian Government should provide life insurance cover with critical illness and total temporary disability for healthcare workers in Nigeria with a sum assured of not less than 25,000,000 Naira per life (65,000 US dollars); provide adequate supplies in terms of social care, stationery and laboratory supplies, increase COVID-19 testing centres and community based learning programmes. Other respondents suggested that the Federal Government should provide charitable donations for the poor, aged, and vulnerable in various communities, including provision of food.

Some respondents thought that healthcare personnel handling HIV antiretroviral therapy (HIV ART) should be trained in COVID-19 testing, with linkage to care and information management for reference laboratories. Respondents also felt that public education on preventive measures should be improved, while others suggested relaxation of the lockdown to allow socioeconomic and agribusiness enterprises to develop. The public health communication process, management of COVID-19 isolation centres, treatment of COVID-19 patients in the community and the enforcement of “stay at home” policies all came under some criticism from respondents.

In summary, for the majority of the respondents:

**“Nigeria had done well”:** some respondents believed that the Federal Government of Nigeria and NCDC had done well and should be commended. As an ostensibly religious nation, a good percentage of the respondents believed that the successes recorded were because of the will of God. Many claimed that God prevented spread of disease, minimized deaths and improved recovery rates. One said, “Thanks to God, NCDC has done very well.” Another was more reflective on leadership and said “Nigerians need to be appreciative of leadership and what they can contribute to the system rather than condemning others.” However, it was felt that “the Federal and State Governments should harmonise policies for the safety of all Nigerians.

**“There was a lack of transparency”:** over 40% of all respondents were of the view that there was little or no transparency in the management of COVID-19 by Government and its agencies. Sample comments included “We should be honest in management of national resources”. Another commented “COVID-19 is a viral infection that can be managed very effectively if citizens are educated, as it should not be a death sentence. If handled well, no life should be lost.”

**“Government should have involved all the critical stakeholders like the Nigeria Medical Association from the very beginning (planning stage)”:** it was felt by respondents that local communities should be engaged and enlightened to support the Federal Government to curtail the spread of the epidemic. One said, “Society should be enlightened on how to boost their immunity to fight COVID-19”. One said, “People should be educated to avoid crowded places, particularly markets during lockdown and on relevant issues, not just washing hands and social distancing.”

**“Government should be more organised in the distribution of social care measures to achieve the purpose of “stay at home” policies”:** the majority of the respondents believed that the distribution of social care measures were poorly handled. For instance, one respondent said “The general distribution of aid packages was very poor, as the beneficiaries hardly got anything from the Government.” A few suggested the use of the Central Bank of Nigeria to credit individual accounts, the use of the Independent National Electoral Commission for social care distribution and prioritization of pregnant women, the aged, and the physically disadvantaged in the distribution process. It was felt that social care measures must be inclusive and not politically driven.

**“Government should rise up to their responsibilities”:** several respondents believed that the government had failed the Nigerian people, did not provide good leadership in the fight against COVID-19, delayed closure of the borders, did not properly enforce social distancing and “stay at home” measures, did not educate the general public appropriately to know more about the epidemic and the preventive steps to take, and did not properly equip healthcare facilities, or train healthcare workers to control the outbreak. Respondents therefore called for responsible leadership, proactive and deliberate steps by Government and its agencies towards COVID-19 control and prevention in Nigeria. They also asked for a stronger drive towards the implementation of the policies of sanctioning erring officers and those who take bribes and exploit travelers.

**“Looking inwards to give research a chance to support the outbreak.”:** a number of respondents were of the view that Nigeria should look inwards for solution to COVID-19. They believed that, like in Madagascar, answers could be found in local herbs, local medications and traditional practices. They were not happy with the strategy of Government in adopting everything the WHO recommended and replicating these strategies in Nigeria. Rather, they asked that Government should fund more medical research to develop local solutions to the COVID-19 challenges. In addition, they believed that western solutions should be properly evaluated and validated before their implementation in Nigeria. To this end, they felt that there was a need to develop home-based care policies and guidelines that were purely Nigerian in origin and content.

**“Rebuilding the health care system and improving its infrastructure”:** many respondents believed that the COVID-19 outbreak revealed the failure of the healthcare system and its lack of capacity to handle emergencies, and suggested “The Nigerian healthcare system should be properly funded to ensure effective service delivery.” To them, there were not enough healthcare workers to handle the problem, and those available were not properly trained in pandemic management. Moreover, it was felt that there was not enough PPE, infrastructure and consumables to handle the pandemic. The respondents therefore asked for people investment in healthcare to rebuild the healthcare system for present and future disease outbreaks. In addition, to achieve the set objectives, respondents suggested that the Government should engage professional associations like the Nigerian Medical Association (NMA) in the planning and implementation of the various control measures.

**“Depoliticising the epidemic”:** some respondents believed that the epidemic was politicized. To this end, they asked for depoliticization of the COVID-19 control process. One said, “We may not have the luxury of large isolation centres to cater for all the people who will test positive, but we can make the treatment protocols available in every locality, so that the majority of people can be treated in their homes. This should be possible if testing kits are available for early detection.”

**“Government should have a solid plan for the after-effects of this crisis on the Nigerian economy...”:** as many nations are already going into economic recession, there was a call by respondents for the Government to plan for the future. For instance, one respondent said “A comprehensive and integrated programming on COVID-19 requires economic planning and social planning. This will increase awareness and help in breaking the chain of community transmission.” To this end, respondents called for funding of agricultural practices to improve food security, support of small and medium scale enterprises to prevent massive loss of employment, and economic planning to ensure a better economic climate for private and public sector enterprises. Sample comments included “The Government should learn a lesson from this and further strengthen the healthcare system, while creating and promoting health policies that would enhance the well-being of the entire community.” We should, according to a respondent, “learn lessons from this pandemic for the future. The Government should implement lessons learned.” One said, “They should allowed air transport, but with COVID-19 testing and quarantine measures put in place to check before and on arrival.”

## Discussion

This is the first qualitative study on the Nigerian population assessing the opinion of the general public to the applicability and application of COVID-19 lockdown and social control measures in Nigeria. As such it provides useful information as to the general mood of the Nigerian people a year into the COVID-19 pandemic. In an elegant study from Italy, a questionnaire format was used to assess perceived efficacy of COVID-19 lockdown and social control measures in that country and the psychological and psychosocial variables that could predict behavioral compliance in that population [8]. While our study is more qualitative than quantitative, compared to the Italian study, it may provide public health modelers baseline information on how to assess predicted compliance and how to adapt public health measures accordingly.

Although comprising a minority of respondents, a number of factors were perceived to have been acceptable in COVID-19 policy in Nigeria, including public health communication measures by NCDC and FMOH (28.0%), enforcement of “stay at home” policies and the use of face-masks (10.3%), the activity of the Office of the Federal Nigerian Presidency and of the Government (9.3%), the activity of NCDC and other healthcare organizations (7.4%), maintained individual interest and attitude to COVID-19 policies (6.4%), management of treatment at medical facilities and isolation centres by medical personnel (5.6%), and feedback from Government in the traditional media (2.5%), all of which received some positive responses. Although these positive responses were few, these participants stated that these were the particular activities that helped control and minimize the spread of COVID-19 in Nigeria. However, some respondents did not believe much that the Government was doing, and felt that it was taking highhanded and unilateral decisions without proper recourse to public opinion. Many participants were not happy with the way COVID-19 policy management had been handled and called for the removal of the team working on COVID-19 and their strategies.

Low COVID-19 testing rates, inadequate facilities, a lack of enforcement of movement restriction, profiting from COVID-19 by media and other stakeholders, the nonenforcement of interstate travelling by security agencies, lack of trust for local drugs, violation of lockdown rules by Nigerians, hunger increase, and ineffective distribution of relief materials were some of the things that were perceived to be lacking by the respondents.

## Conclusion

COVID-19 has resulted in a global great paradigm shift. Three important priorities set the pace for a post-COVID-19 reality check and these are: planning for future pandemics through the procurement and supply of essential healthcare products, including PPE and specific training of frontline medical personnel; strengthening crisis management and response through harmonisation of regional pandemic management committees to be coordinated at a national level and greater provision of healthcare funding for public health awareness campaigns. As economic restrictions are gradually eased to reopen personal and business activities, the possibility of facing public disquiet over persistent policies, such as social distancing in the *“The New Normal”* are a real possibility unless mass education is undertaken at all levels from radio, television and importantly in 2021, social media platforms. Although difficult to extrapolate findings from other countries, as commonly held beliefs vary from country to country [9], a recent Italian questionnaire held that young people were more likely to comply with social control measures if they had incorporated physical activity of some form into their daily lives at home during the lockdown period. Future Nigerian questionnaires should look at this issue acting as a mitigating factor in the general public dissatisfaction that we have found in our survey, given that exercise alleviates boredom and provides a sense of well-being, even during lockdown [10].

Steps to return to a robust economy in the peri-COVID era would include maintained hand-washing, social distancing and limited group contact until COVID-19 vaccines are widely available in Nigeria. We advocate coordinated forward planning for public safety until the concept of vaccination is widely accepted by the Nigerian general public, rather than being viewed with suspicion. Policymakers need to be adaptable to rapidly changing conditions, given fluctuating case numbers and mounting fatality rates. The question of international travel is also an issue for policymakers, given that people these days have been used to freedom to travel wherever they choose. However, it is known from other viral diseases that travel serves to spread cases around the world, and COVID-19 had been no different in this respect [1]. Public opinions on limiting international travel also need to be assessed in the Nigerian context in future studies.

A limitation of the study is the potential lack of representatives with respect to the Nigerian population as a whole. The questions were phrased in English and this excluded non-English speakers. Furthermore, Table 1 shows that a high proportion of the respondents had received tertiary education and were in employment. It could be argued that the results reflect the opinions of a wealthy politically aware elite, rather than being opinions of the whole population. However, although this study was of small sample size, it was open for the participants to access on mobile phone (ownership of which is widespread in Nigeria) or on computer. We admit that by its study design, the questionnaire was always likely to have attracted the better educated and computer literate members of society and possibly an undue number of people with political axes to grind, but we feel it is a reasonable reflection of how certain sections of Nigerian society has been feeling during the COVID-19 lockdown process.

### What is known about this topic

- COVID-19 pandemic has led to a complete shift in how pandemics are managed across the world;
- Nigerian government through the Federal Ministry of Health, Nigerian Center for Disease Control (NCDC) and Presidential Task Force on COVID-19 implemented several strategies to curtail the impact of the pandemic;
- Daily epidemic reports were provided to Nigerians and the rest of the world detailing efforts made, cases identified and mortalities recorded.

### What this study adds

- Provides insight into the perspective of Nigerian stakeholders on the activities, polices and practices of government and her agencies in relation to the COVID-19 outbreak and control;
- Gives suggestions to government and her agencies on how best to manage future epidemics/pandemics for improved health outcomes thereby minimizing morbidities and mortalities from epidemic;
- Suggests the need to involve relevant and critical stakeholders early in the pandemic management process.
